# Assessing the Psychometric Properties of the Internet Addiction Test (IAT) Among Lebanese College Students

**DOI:** 10.3389/fpubh.2018.00365

**Published:** 2018-12-17

**Authors:** Ali A. Samaha, Mirna Fawaz, Najwa El Yahfoufi, Maya Gebbawi, Hassan Abdallah, Safaa A. Baydoun, Ali Ghaddar, Ali H. Eid

**Affiliations:** ^1^Doctoral School for Human and Social Sciences, Lebanese University, Beirut, Lebanon; ^2^Faculty of Health Sciences, Beirut Arab University, Beirut, Lebanon; ^3^Department of Biomedical Sciences, Lebanese International University, Beirut, Lebanon; ^4^Rayak University Hospital, Rayak, Lebanon; ^5^Department of Pediatrics and Adolescents Medicine, American University of Beirut, Beirut, Lebanon; ^6^Research Center for Environment and Development, Beirut Arab University, Bekaa, Lebanon; ^7^Department of Pharmacology and Toxicology, American University of Beirut, Beirut, Lebanon

**Keywords:** internet addiction test, psychometrics, addictive behavior, internet, Lebanon

## Abstract

Internet addiction is an emergent problem; yet, both a strong conception of the factors precipitating challenging activities and a gold standard tool for evaluating symptoms are deficient. The aim of this study was to carry out a psychometric analysis using the most commonly employed screening tool, the young Internet Addiction Test (IAT), comprising a sample of Lebanese University medical students. Two hundred and fifty-six undergraduate medical students from a university in Beirut, Lebanon were included in our IAT. Exploratory factor analysis was employed, and four factors were extracted. These four factors were named as Lack of Control, Social Withdrawal and Emotional Conflict, Time Management Problems, and Concealing Problematic Behavior. Furthermore, the selected factors explained 56.5% of the total variance. Cronbach's alpha coefficient for the internal reliability of the scale was found to be 0.91. For each subscale, the internal consistency score was approximated and detected as 0.76, 0.74, 0.69, and 0.63 for the first through fourth factor, respectively. Item total correlations were calculated and had a value range from 0.37 to 0.63 for the 20 items. IAT is a proper tool for evaluating internet addiction in Lebanese college students.

## Introduction

The significant existence of internet in our communities has created unease over the potential presence of an internet addiction condition. While this outlook might be debatable, the future incorporation of internet addiction in the Diagnostic and Statistical Manual of Mental Disorders-V as a disorder requires additional research and further evaluation and exploration (1). At its quintessence, internet addiction is delineated by repeated, uncontrolled and risky use of the internet. Internet addiction should not be mystified with gaming disorder which is a diagnosis characterized by the disobedient and relentless playing of video and computer games, which is hurtful to an individual's well-being (2). The Symptoms of internet addiction that are usually detected in clinical environments include obsession, withdrawal, and lack of control and performance deficiency (3). Improving our perception of how these symptoms delineate and incentivizes internet addiction will aid in shaping this evolving area of research and consequently, in improving ways to manage or treat it (4). According to the Internet World Statistics, there was an approximated sum of 5,546,494 internet users in Lebanon as of December 31, 2017 (5). The documented penetration rate in Lebanon is 91.0 and 64.5% in the Middle East. Lately, and due to the severity of this addiction, the Lebanese Ministry of Telecommunication has granted access to researchers tackling internet addiction in Lebanon (6). Indeed, the penetration rate is relatively high compared to neighboring countries, where the penetration ranges from 24.3% in Yemen to 98.4% in the United Arab Emirates (5).

Developing a consistent tool for examining internet addiction indications in clinical and research backgrounds is a much needed step. Of the accessible tools, the most commonly employed method is the Internet Addiction Test (IAT). Primary examination into the validity of the IAT has shown strong internal consistency (α = 0.90–0.93) and good test-retest reliability (*r* = 0.85) values (7–12).

Several instruments for Internet addiction estimation have been developed, but none have emerged as the “gold standard” (13). The most commonly used ones are the Internet addiction test (IAT), the Young of the Internet Addiction Questionnaire (YDQI), the Chen's Internet addiction scale (CIAS) and the Internet addiction scale (IAS). The Internet Addiction Test (IAT) was created by Young (1998) to evaluate the existence and intensity of internet addiction in a North American population sample (14). The tool encompasses various internet use demeanors and recurrent addiction indicators, with the noteworthy exclusion of tolerance. The instrument comprises 20 items; each was extracted from previous studies and clinical research on obsessive online consumers and their features. These 20 elements evaluate attributes and demeanors related to obsessive use of the internet that comprises escapism, compulsivity, and dependency. The inquiries also examine conflicts in personal, social, or occupational performance that may stem from addictive use (15). Importantly, these questions are randomized with each statement scored on a Likert-scale with values ranging from 0, indicating less radical behavior, to 5 indicative of the most radical behavior for each item. The test could be applied either on individual basis or in a collective sample. It can be applied in two methods: self-administered and verbally, if anyone required help in fulfilling the questionnaire. When self-administered, the rest requires 5–10 min to fill (15). Each item on the questionnaire is equally valued on a 5-point scale, with the total maximum score being 100. A higher score signifies a higher level of intensity in internet obsession and addiction. A total score that does not exceed 30 indicates a normal level of internet consumption, whereas total scores between 31 and 49 indicate mild level addiction, 50–79 designates moderate addiction, and scores of 80 or above reflect a severe internet dependency (15).

Behavioral addictions usually occur during teenage years or young adulthood (16). University students, are especially susceptible to internet addiction (17). University students account for near global rates of computer acquisition, Internet availability and day to day consumption; the majority devote at minimum 2 h online everyday (18–20). The frequency of internet addiction is usually documented to be augmented in the midst university students than different teenage populations (21–25). Among US college undergraduates, the frequency of internet addiction is ~8–25% (26–31), a percentage close to that of substance use conditions or uncontrolled gambling (32, 33). Given the increased predisposition for challenging conduct or behavior within this subgroup, the development of a consistent tool for evaluation is immensely necessitated.

Studies investigating internet addiction among medical students in Lebanon are deficient. Hence, this study was undertaken to examine the construct validity of the IAT in an at-risk, college student Lebanese population. By carrying out a factor analysis of the IAT, we seek to (1) investigate the behavioral elements causing addictive internet use, and (2) evaluate the validity of the IAT as a screening tool particularly in this Lebanese population or similar at-risk populations.

## Methods

### Subjects

A sample of medical students enrolled in a private university in Lebanon adopting the French teaching system/curriculum were selected. Medical students were chosen since they are considered very frequent users of internet. Undergraduate medical students aged 18–29 from different academic years were enrolled in this study. Although students in different years may be expected to spend different times using the internet, the academic level was neglected since the study focus on assessment of psychometric properties and validity of an assessment tool. Eligible participants were identified as being enrolled in the medical school at the mentioned university. IRB approval was obtained from the Lebanese University, Doctoral School for Social Sciences and Literature (Approval Number: PS 2017).

### Data Collection

A cross-sectional research design was adopted including of 256 medical students from a private university in Lebanon. The study was carried out in September 2017 to obtain the maximum number of respondents since September marks the beginning of the acemidc year and students are relatively not stressed by exams and workload. Students were given short instructions on the self-administered Internet Addiction Test (IAT) (14). Survey elements comprised of an informed consent sheet, socio-demographic questionnaire and the IAT. The IAT comprises twenty elements evaluating a person's efficiency in their school, occupation, home (3 questions), social demeanor (3 questions), emotional relationship with and reaction due to use of the internet (7 questions), as well as internet usage patterns (7 questions). Participants filled all the IAT items on a 6-point Likert measure (“does not apply” to “always”), which sums up to total score range from 0 to 100.

### Statistical Analysis

Statistical analyses were performed using SPSS version 24. Individual item scores of IAT and demographic characteristics were summed up using descriptive analyses.

Exploratory factor analysis were carried out to evaluate the construct validity of IAT. Visual evaluation of a scree plot in conjunction with the orthodox cut-off of eigenvalues < 1 were used to determine the number of factors to be extracted. In order to identify independent underlying constructs and factor loadings, Varimax rotation was employed. Items were allocated to the factor which generated the highest factor loading. By approximating Cronbach's alphas, the internal consistency of each factor was established. In order to confirm the degree of independence between the factors recognized, we then evaluated the linear correlation coefficient between factors.

## Results

### Descriptive Statistics

The participants in this study were 36.6% males and 63.4% females and their ages ranged from 18 to 29 years of age (Mean = 21.92, SD = 2.16), and their GPAs ranged from 0.55 to 4.00 (Mean = 3.00, SD = 0.52). 31.3% of the participants scored below 30 on the total Internet Addiction Scale signifying normal use of the internet. 38.2% of the participants scored between 31 and 49 on the total Internet Addiction Scale signifying mild addiction level. 28.9% scored between 50 and 79 on the total Internet Addiction Scale that signifies a moderate level of internet addiction, while 1.2% of the participants scored above 80% signifying severe dependence upon the internet.

### Exploratory Factorial Analysis (EFA)

In the first phase of the study, an exploratory factor analysis with 256 university students was run. Maximum likelihood extraction method and Varimax rotation technique were used because these techniques are known to provide a good estimation of a many indexes regarding the goodness of fit of the model as well as allows for the determination of statistical analysis of factor loadings and correlations among factors in addition to providing a reliable computational determination of confidence intervals (34). EFA showed four factors with eigenvalues more than 1. These four factors along with corresponding items and factor loadings are displayed in Table 1. The factor loads associated with the 20 items on a scale ranged from 0.440 to 0.769. Thus, it was determined that these questions were sufficiently qualified to be incorporated into the test. Items 11, 12, 13, 16, and 17 were in the first factor; items 15, 20, 14, 18, 19, 4, and 3 in the second factor; items 2, 1, 5, 8, and 6 in the third factor; and items 10, 7, and 9 in the fourth factor. These four factors explained 56.5% of the total variance (see Table 2).

**Table 1:** Factor loading resulted from EFA analysis.

**Item**		**F1**	**F2**	**F3**	**F4**
Q16	Do you find yourself saying “just a few more minutes” when online?	**0.713**	0.043	0.403	0.083
Q12	Do you fear that life without the Internet would be boring, empty or joyless?	**0.663**	0.181	0.134	0.223
Q17	Do you try to cut down the amount of time you spend online?	**0.622**	0.017	0.341	0.268
Q13	Do you snap, yell or act annoyed if someone bothers you while you are online?	**0.589**	0.492	0.027	0.128
Q11	Do you find that you find yourself anticipating when you will go online again?	**0.467**	0.331	0.230	0.324
Q15	Do you feel preoccupied with the Internet when offline, or fantasize about being online?	**0**.500	**0.540**	0.159	0.015
Q20	Do you feel depressed, moody or nervous when you are offline, which goes away when you are back online?	0.495	**0.546**	0.097	0.120
Q14	Do you lose sleep due to late night log-ins?	0.410	**0.453**	0.435	−0.029
Q18	Do you try to hide how long you've been online?	0.386	**0.440**	0.153	0.362
Q19	Do you choose to spend more time online over going out with others?	0.183	**0.708**	0.213	0.215
Q4	Do you form new relationships with fellow online users?	0.064	**0.604**	0.243	0.023
Q3	Do you prefer the excitement of the Internet to intimacy with your partner?	0.005	**0.636**	0.022	**0**.319
Q2	Do you neglect household chores to spend more time online?	0.398	0.066	**0.542**	0.191
Q1	Do you find that you stay online longer than you intended?	0.334	−0.054	**0.691**	0.025
Q5	Do others in your life complain to you about the amount of time you spend online?	0.187	0.339	**0.572**	0.188
Q8	Does your job performance or productivity suffer because of the internet?	0.050	0.272	**0.646**	**0**.343
Q6	Do your grades or schoolwork suffer because of the amount of time you spend online?	0.100	0.372	**0.735**	0.172
Q10	Do you block out disturbing thoughts about your life with soothing thoughts of the internet?	0.204	0.326	0.199	**0.599**
Q7	Do you check your email before something else that you need to do?	0.102	−0.001	0.138	**0.769**
Q9	How often do you become defensive or secretive when anyone asks you what you do online?	0.289	0.294	0.171	**0.612**

*Bold values indicate values greater than the Factor loading cut-off point set at 0.4*.

**Table 2:** Variance explained by the factors.

**Factor**	**Variance**	**% of Variance**	**Cumulative %**
1	3.202	16.008	16.008
2	3.172	15.861	31.869
3	2.847	14.235	46.103
4	2.089	10.447	56.550

This variance rate implied that this test can be evaluated as a test composed of four factors, namely Lack of Control, Social Withdrawal and Emotional Conflict, Time Management Problems, and Concealing Problematic Behavior. In the Lack of Control factor, questions mostly revolved around preoccupation with internet consumption. In the Time Management Factor, questions tackled fulfilling duties and responsibilities. In the Social Withdrawal and Emotional Conflict factor, questions assessed emotional states and relationships with others. Finally, in the Concealing Problematic Behavior factor, questions were pertinent to hiding internet problem and pattern of consumption.

### Confirmatory Factorial Analysis (CFA)

**Figure 1:**
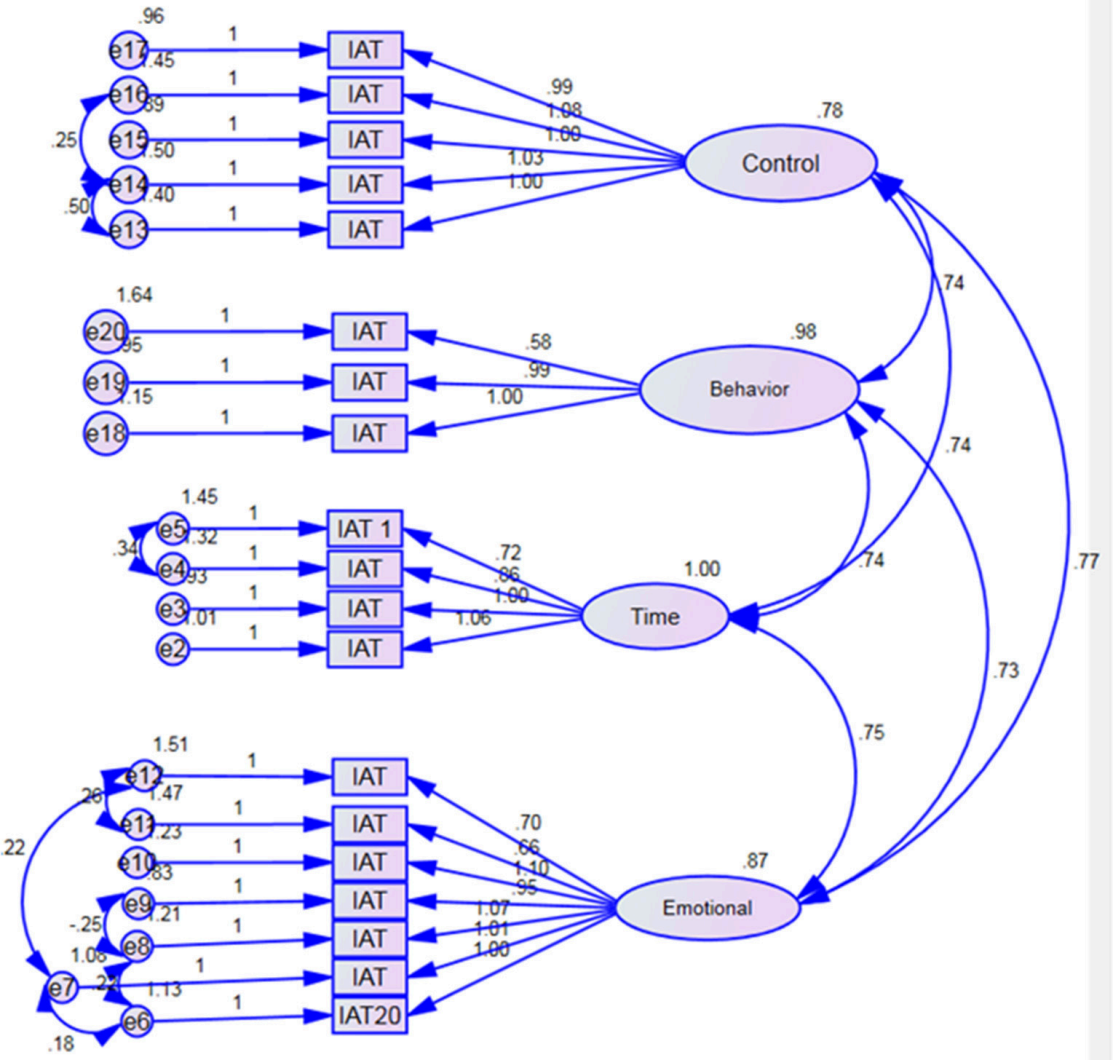
Confirmatory factorial analyses for internet addiction test components.

In the next phase, a CFA was carried out to ratify IAT's factor structure that was revealed in the EFA. CFA analysis was carried out with another sample. CFA's first run resulted in χ^2^ (df = 164, *p* < 0.001) = 446.520 and χ^2^/df = 2.72. These estimates are relative to sample size, specifically in models with large number approximated parameters. There were other indexes that were calculated such as: Comparative Fit Index (CFI) = 0.875, Normed Fit Index (NFI) = 0.818, Goodness of Fit Index (GFI) = 0.867, and Root Mean Squared Error of Approximation (RMSEA) = 0.076. It is known that when the values of CFI, NFI, and GFI exceed 0.90, and that of RMSEA is < 0.08, then the construct is considered acceptable (35, 36). Importantly, in our current model, the values of NFI and GFI did not exceed 0.90. This prompted us to check if the modification indices in the result of the CFA for any proposed covariance. Then, separate covariance between the errors e4-e5, e13-e14, e15-e16, and e11-e12, e12-e7, e8-e9, and e6-e7 were created, respectively. In addition a1, was excluded as it scored a residual covariance higher than absolute 2.58 on multiple elements. The CFA analysis was rerun, and χ^2^ (df = 138, p < 0.001) = 297.882 and χ^2^/df = 2.15 were observed. Other fit indexes, including CFI = 0.923, NFI = 0.90, GFI = 0.906, and RMSEA = 0.062 were described to assess the model fit. The values of CFI, NFI, and GFI exceeded 0.90, and that of RMSEA was found to be < 0.08; therefore, the construct is considered acceptable. Hence, the CFA findings in our current approach argue that the model is coherent. As shown in Figure 1, all factor loadings were found to be statistically significant (*p* < 0.05). Table 3 shows both the inter-factors as well as factor-total point correlations. As can be seen, all correlations were found to be positive as well as statistically significant.

**Table 3:** Inter-item correlation matrix.

	**F1**	**F2**	**F3**	**F4**
F1		0.697^*^	0.643^*^	0.566^*^
F2			0.616^*^	0.559^*^
F3				0.533^*^
F4				

* *indicates significant values in the inter-item correlation matrix that are higher than 0.5*.

In regards to the reliability of the measurement, Cronbach's alpha coefficient was estimated. The internal reliability score of the scale was 0.914. Moreover, the internal consistency score for each subscale were determined. These values were 0.76, 0.74, 0.69, or 0.63 for the first, second, third or fourth factor, respectively. Values for item-total correlations ranged from 0.38 to 0.64 for the 20 tested items.

## Discussion

The primary objective of this research was to investigate the psychometric properties of IAT. It first examined the 5-point rating scale. The extracted components highly explained the questionnaire which was consistent with Panayides and Walker (37) who reported total variance of 40.8% with an eigenvalue of 13.8 for principal component analysis (PCA). They argued for a unidimensional structure of IAT. Our results reported a higher variance explained by the measure of 20-item IAT (56.5%, of the total variance). Our study, thus, further reinforces the unidimensional structure of IAT.

Previous research has revealed one- to four-factor model for IAT; thus, we undertook this research to investigate the one- to four-factor solution utilizing EFA and CFA, respectively, and showed that the four-factor model was fit. The various factor models revealed in previous studies or in this report may be affected by the varying cultures and sample backdrops. Widyanto and McMurran (7) first recognized a six-factor solution amongst UK university students, and another six-factor solution was distinguished by employing a more discrete age group in an Italian population with the age of participants ranging from 13 to 50 years old (38). Interestingly, a three-factor solution was recognized for a Chinese sample in the bilingual (English and Chinese) version IAT (9, 39). With disregard to the various factor distribution among the four-factor model in this and previous reports, the key underlying structure of IAT remains consistent. For example, F1 (lack of control) in this study was very analogous to F1 in a study involving medical students in Malaysia (40). F2 in this study, designated as social withdrawal and emotional conflict, was similar to Factor of Mood in a four-factor model (41). F3 in this study, designated as Time Management Problems, was similar to Responsibility factor in a four-factor model (41) and to Time Management and Performance Factor in two other studies (9, 39). Finally, F4 of this study, labeled as Concealing Problematic Behavior, was not consistent with what is reported in the literature. In contrast to the former four-factor models, this study recognized IAT97, IAT9, and IAT 10 as one factor labeled deliberately (concealing problematic behavior). IAT 9 is “defensive or secretive when anyone asks you what do you online,” while IAT 7 is “do you block out disturbing thoughts about your life with soothing thoughts of the internet?” Both elements in IAT are associated with hiding the real internet consumption of the individual involving content and duration spent. Former studies distributed these two elements in “factor of mood” in a four-factor solution (41), “neglect of duty” factor of five-factor solution (40), “withdrawal & social problem” factor of a three-factor solution (9, 39), “psychological/emotional conflict” factor of a three-factor model (7), “salient use” factor of a two-factor model (12), “dependent use” factor of a two-factor model (42). Even though there was a relationship between these two elements and the factors enumerated above, IAT 9, IAT 7, and IAT 10 were evidently gathered as a factor of “deliberately concealing problematic behavior” in this study. A contemporary research for a Malay version IAT defined five factors among medical students (neglect of duty, social relationship disruption, lack of control, problematic use, and email privacy) (40). One of the factors, email privacy, was only founded by one item IAT7, which was recommended to be dropped.

The psychometric properties of the original IAT were examined in university students and adults. A strong internal reliability estimates of IAT has been suggested; yet, there was a discrepancy amongst the described factor structures of IAT. Widyanto and McMurran (7) carried out a research amongst adults in the U.K. and an exploratory factor analysis (EFA) exposed six factors to be intimately interweaved with IAT. These factors were neglect of work, salience, anticipation, excessive use, lack of control, and neglect of social life. Another research enrolled university students in the United Kingdom (U.K.) carried out by Widyanto, Griffiths, and Brunsden (43) revealed a 3-factor solution, which involved psychological/emotional discord, time management conflicts, and mood modification. In a more contemporary research, a two-factor solution of the IAT was revealed among U.S. university students (42). These factors were dependent use and excessive use. The IAT was also employed in studying different populations including French (10), Italian (38), Finnish (12), Korean (44), Malay (40), and Chinese (9) university students and adults. In the Italian study, six-factor solution (compromised academic/working careers, compromised social quality of life, compensatory usage of the internet, compromised time control, compromised individual quality of life, and excitatory use) was found. A one-factor solution in the French and Finnish versions and five factors (lack of control, neglect of duty, problematic use, social relationship disruption, and email primacy) in the Malay version were reported. These diverse results on the psychometric validation of the IAT have designated the irregularities on the factor structure of the original tool. In accordance with Lai et al. (39), these variations could be due to differences in languages, demographics of the samples, and statistical techniques being employed. Previous research also steadily displayed high reliability estimates of the IAT, with an α > 0.80 (11, 45, 46).

Young recommended six dimensions in the initial version of the IAT (14): Salience (item 10, 12, 13, 15, and 19) associated that “respondent most likely feels preoccupied with the internet, hides the behavior from others, and may display a loss of interest in other activities and/or relationships only to prefer more solitary time online,” excessive use (item 1, 2, 14, 18, and 20) associated that “respondent engages in excessive online behavior and compulsive usage, and is intermittently unable to control time online that he or she hides from others,” neglect work (item 6, 8, and 9) associated that respondents' “performance and productivity are most likely compromised due to the amount of time spent online and the respondent may become defensive or secretive about the time spent online,” anticipation (item 7 and 11) related that “respondent most likely thinks about being online when not at the computer and feels compelled to use the internet when offline,” lack of control (item 5, 16, and 17) related that “respondent has trouble managing his or her online time, frequently stays online longer than intended, and others may complain about the amount of time he or she spends online,” and neglect social life (item 3 and 4) related that “respondent most likely utilizes online relationships to cope with situational problems and/or to reduce mental tension and stress.”

Additional evaluation of the IAT is recommended for both theoretical and psychometric purposes. Primarily, the IAT was developed as a unidimensional tool with each item similarly weighed to add up to the overall score. Yet, previous studies have displayed an ambiguous factor structure; Table 4 offers a sum up of these results. Second, to the best of our knowledge, the psychometric properties of the IAT have not been evaluated in a Lebanese population. As cultural variances may influence the appearance of problematic internet use behaviors, results issued from international communities may not be relevant to Lebanese populations.

**Table 4:** Factor structure of IAT in the prior research.

**Model**									
**Item**	**1a/b**	**2a**	**2b**	**3a**	**3b**	**3c**	**5**	**6a**	**6b**
IAT1	1	2	2	2	2	2	1	2	5
IAT2	1	2	2	2	2	2	1	2	2
IAT3	1	1	1	1	1	1	2	6	6
IAT4	1	1	1	3	1	1	3	6	1
IAT5	1	1	1	1	1	1	4	5	1
IAT6	1	2	1	2	2	2	2	3	4
IAT7	1	2	2	2	-	-	5	4	3
IAT8	1	2	1	1	2	2	2	3	4
IAT9	1	1	1	1	1	1	2	3	1
IAT10	1	1	1	1	3	3	1	1	6
IAT11	1	1	1	1	-	-	1	4	3
IAT12	1	1	1	3	3	3	1	1	2
IAT13	1	1	1	3	1	1	4	1	1
IAT14	1	2	1	3	3	3	1	2	2
IAT15	1	1	1	3	1	1	3	1	3
IAT16	1	2	2	2	2	2	1	5	1
IAT17	1	2	2	1	2	2	1	5	5
IAT18	1	1	1	1	1	1	2	2	1
IAT19	1	1	1	1	1	1	2	1	2
IAT20	1	1	1	3	1	1	2	2	2

*1a: derived from Khazaal et al. (10) (EFA andandand CFA)*.

*1b: derived from Korkeila et al. (12). EFA*.

*2a: F1— dependent use; F2—excessive use. Fioravanti and Casale (42). EFA*.

2b: F1—salient use; F2—loss of control. Korkeila et al. (12). EFA

3a: F1—psychological/emotional conflict; F2—time-management problems; F3—mood modification. Widyanto et al. (43). EFA

3b: F1—withdrawal andandand social problems; F2—time management andandand performance; F3—reality substitute. Chang and Law (9). EFA andandand CFA z 3c: F1—withdrawal andandand social problems; F2—time management andandand performance; F3—reality substitute Lai et al. (39). CFA

5: F1—lack of control; F2—neglect of duty; F3—problematic use; F4—social relationship disruption; F5—email privacy. Guan et al. (40). EFA

6a: F1—salience; F2—excessive use; F3—neglect work; F4—anticipation; F5—lack of control; F6—neglect social life. Widyanto and McMurran (7). EFA

6b: F1—compromised social quality of life; F2—compromised individual quality of life; F3—compensatory usage of the Internet; F4—compromised academic/working careers;

*F5—compromised time control; F6—excitatory usage of the Internet. Ferraro et al. (38)*.

## Conclusion and Recommendations

Even though this research examined the structure of IAT using EFA, the differences obtained in comparison to other four-factor solutions or other five and six factor models may be related to the varying approximation techniques and rotation type for EFA, as well as the sample composition such as differences in the cultures of the sample.

In this study, our targeted population was university students. Therefore, generalizing findings to other teenage or young adult populations might not be recommended. Knowing that university students are an important population in which increased internet consumption is usual and possibly required, this was our population of choice for this study. It is warranted that our results shall be analyzed and explained with some attention, as findings may have been influenced by features related to a campus culture and environment. In specific, participants' displaying extreme usage signs may have been increased in the setting of high standards of internet consumption reported among students. If this were indeed the case, it would propose that when creating methods for screening, diagnosing or managing internet addiction, special attention should be taken to incorporate clinical strategies along with the technology consumerism standards related to the sample of interest.

Despite these drawbacks, our findings provide an exciting insight that warrants further studies regarding the IAT and internet addiction. While our results adopt the IAT as a useable evaluation of internet addiction in the studied population, further confirmatory analysis of our two-factor model is essential to ratify these findings. Moreover, the simultaneous and prognostic validity of the various scores (overall cut-offs, item-weighing scheme or sub-scale scores) should be examined by employing clinical assessments. These will be demanding tasks, given the deficiency of gold-standard actions for internet dependency and unwarranted internet consumption. Finally, further work is required to conclusively establish whether internet addiction signs can be safely projected onto other pertinent populations such as individuals with current psychiatric illness or those displaying more drastic addiction symptoms.

## Author Contributions

AS and AE conceived of the project. AS, MF, and AE designed the work. MF, NE, MG, HA, SB, and AG collected and analyzed the data. MF and AE wrote the manuscript.

### Conflict of Interest Statement

The authors declare that the research was conducted in the absence of any commercial or financial relationships that could be construed as a potential conflict of interest. The reviewer KZ declared a shared affiliation, with no collaboration, with one of the authors, NE, to the handling editor at time of review.
